# Clinical trial of insulin-like growth factor-1 in Phelan-McDermid syndrome

**DOI:** 10.1186/s13229-022-00493-7

**Published:** 2022-04-08

**Authors:** A. Kolevzon, M. S. Breen, P. M. Siper, D. Halpern, Y. Frank, H. Rieger, J. Weismann, M. P. Trelles, B. Lerman, R. Rapaport, J. D. Buxbaum

**Affiliations:** 1 Seaver Autism Center for Research and Treatment, New York, NY USA; 2 Friedman Brain Institute, Icahn School of Medicine at Mount Sinai, New York, NY USA; 3 Mindich Child Health Institute, Icahn School of Medicine at Mount Sinai, New York, NY USA; 4 Department of Psychiatry, Icahn School of Medicine at Mount Sinai, New York, NY USA; 5 Department of Pediatrics, Icahn School of Medicine at Mount Sinai, New York, NY USA; 6 Department of Neuroscience, Icahn School of Medicine at Mount Sinai, New York, NY USA; 7 Department of Neurology, Icahn School of Medicine at Mount Sinai, New York, NY USA; 8 Department of Genetics and Genomic Sciences, Icahn School of Medicine at Mount Sinai, New York, NY USA; 9 Department of Endocrinology and Diabetes, Icahn School of Medicine at Mount Sinai, New York, NY USA; 10 Icahn School of Medicine at Mount Sinai, One Gustave L. Levy Place, Box 1230, New York, NY 10029 USA

**Keywords:** Phelan-McDermid syndrome, PMS, shank3, Autism spectrum disorder, ASD, Insulin-like growth factor-1, IGF-1

## Abstract

**Background:**

Phelan-McDermid syndrome (PMS) is caused by haploinsufficiency of the *SHANK3* gene and is characterized by global developmental delays and autism spectrum disorder (ASD). Based on several converging lines of preclinical and clinical evidence supporting the use of insulin-like growth factor-1 (IGF-1) in PMS, this study aims to follow-up a previous pilot study with IGF-1 to further evaluate this novel therapeutic for core symptoms of ASD in children with PMS.

**Methods:**

Ten children aged 5–9 with PMS were enrolled. Participants were randomized to receive IGF-1 or placebo (saline) using a 12-week, double-blind, crossover design. Efficacy was assessed using the primary outcome of the Aberrant Behavior Checklist—Social Withdrawal (ABC-SW) subscale as well as secondary outcome measures reflecting core symptoms of ASD. To increase power and sample size, we jointly analyzed the effect of IGF-1 reported here together with results from our previous controlled trail of IGF-1 in children with PMS (combined N = 19).

**Results:**

Results on the ABC-SW did not reach statistical significance, however significant improvements in sensory reactivity symptoms were observed. In our pooled analyses, IGF-1 treatment also led to significant improvements in repetitive behaviors and hyperactivity. There were no other statistically significant effects seen across other clinical outcome measures. IGF-1 was well tolerated and there were no serious adverse events.

**Limitations:**

The small sample size and expectancy bias due to relying on parent reported outcome measures may contribute to limitations in interpreting results.

**Conclusion:**

IGF-1 is efficacious in improving sensory reactivity symptoms, repetitive behaviors, and hyperactivity  in children with PMS.

*Trial registration* NCT01525901.

**Supplementary Information:**

The online version contains supplementary material available at 10.1186/s13229-022-00493-7.

## Introduction

Mutations in distinct risk genes are now understood to contribute to autism spectrum disorder (ASD). *SHANK3* is one important example; *SHANK3* codes for a critical scaffolding protein in the postsynaptic density of glutamatergic synapses [1]. *SHANK3* haploinsufficiency causes Phelan-McDermid syndrome (PMS) [2, 3], a common cause of ASD [4]. This study is the second of two projects examining the use of insulin-like growth factor-1 (IGF-1) as a novel treatment for PMS.Insulin-like growth factor-1 (IGF-1) is a commercially available compound that crosses the blood–brain barrier [5] and has beneficial effects on synaptic maturation and plasticity [6]. There are now several converging lines of evidence to support the use of IGF-1 in PMS based on results from a *Shank3*-deficient mouse model [7], neurons derived from patient derived pluripotent stem cells [8], and children with PMS [9]. In addition, evidence for the utility of IGF-1 and related compounds has been accumulating in other neurodevelopmental disorders associated with ASD, including Rett syndrome [10, 11] and Fragile X syndrome [12].

The primary aim of this study was to evaluate the safety and efficacy of IGF-1 vs. placebo in children with PMS using the Aberrant Behavior Checklist—Social Withdrawal subscale (ABC-SW) as a primary outcome measure. Our secondary aim was to explore effects on associated symptoms of ASD using measures of sensory reactivity, repetitive behaviors, and other aberrant behaviors.

### Design

Participants were enrolled in two consecutive studies, the first of which was previously published [9]. In both studies, treatment with IGF-1 or placebo was divided into two Phases (1 and 2). Participants were randomly assigned to receive either IGF-1 or placebo for 12-weeks in Phase 1 and were then switched to the other treatment condition (Phase 2) after a four-week wash-out period. The second study was completed in September, 2016 and results are presented herein, along with results combining the two studies.

### Participants

The first study screened and enrolled nine children with PMS (6 females and 3 males) aged 5 to 15 years old (mean = 8.6; SD = 4.0) [9]. This second study screened 11 children and enrolled 10,one dropped out during the screening procedures. Participants were between 5 and 9 years old (mean = 6.5; standard deviation = 1.4); 6 participants were male and 4 were female. Nine of 10 participants met criteria for ASD based on clinical consensus using the Autism Diagnostic Observation Schedule, Second Edition [13], the Autism Diagnostic Interview-Revised [14], and the Diagnostic and Statistical Manual for Mental Disorders, Fifth Edition [15].

### Inclusion criteria

Participants were required to have pathogenic deletions or sequence variants of the *SHANK3* gene for inclusion: six had terminal deletions and four had sequence variants. All participants were required to be on stable medication regimens for at least three months prior to enrollment.

### Exclusion criteria

Potential participants were excluded if any of the following were applicable: (1) closed epiphyses; (2) active or suspected neoplasia; (3) intracranial hypertension; (4) hepatic insufficiency; (5) renal insufficiency; (6) cardiomegaly/valvulopathy; (7) allergy to IGF-1; (8) patients with comorbid conditions deemed too medically compromised to participate.

### Drug administration

IGF-1 is an aqueous solution for injection containing human insulin-like growth factor-1 (Increlex; Ipsen Biopharmaceuticals, Inc) produced by recombinant DNA technology. Placebo was normal saline prepared in identical bottles by the research pharmacy at the Icahn School of Medicine at Mount Sinai. Dose titration was initiated at 0.04 mg/kg twice daily by subcutaneous injection, and increased, as tolerated, every week by 0.04 mg/kg per dose to a maximum of 0.12 mg/kg twice daily. Medication was administered subcutaneously twice daily with meals and glucose monitoring was performed by parents prior to each injection and at bedtime.

### Safety

Participants underwent comprehensive medical evaluations, including physical and neurological examination, routine hematology and blood chemistry, bone X-ray for bone age, electrocardiography, and echocardiography to determine eligibility for participation and repeated throughout the study to assess safety. Tolerability was monitored using a safety monitoring report form. Patients were monitored at weeks 2, 4, 6, 8, and 12 in both treatment phases. The most common side effects of IGF-1 are related to its insulin-like activity and hypoglycemic risks. Training was conducted with parents at baseline visits for drawing finger stick blood glucose levels and monitoring for signs and symptoms of hypoglycemia. Training in administering subcutaneous injections was also performed. Hypoglycemia was defined as glucose < 50 mg/dL.

### Efficacy

The clinical outcome measures were administered at baseline and weeks 4, 8, and 12. Measures included the ABC [16], the Repetitive Behavior Scale—Revised (RBS-R; [17]), the Sensory Profile (SP; [18]), and the Clinical Global Impressions—Improvement and Severity Scales (CGI; [19]). Results from the SP and CGI were not previously reported in the first IGF-1 trial.

### Data analysis

All statistical analyses were conducted in the statistical package *R*. Before testing for efficacy, we conducted analyses to test for potential bias in study design. We first performed a pre-test to check the assumption of negligible carryover effects between Phase 1 and Phase 2 of the crossover trial, as previously described [20], to ensure data collection was highly standardized across all patients and that the wash-out period was successful in removing significant carry-over effects. In brief, the sum of the values measured in the two phases was calculated for each subject and compared across the two sequence groups by means of a statistical test for independent samples. This trial used a randomized crossover design; thus, order (phase) of assessment is nested (repeated) within treatment and treatment is nested within subjects. Therefore, in terms of efficacy measurement, we applied a treatment × time interaction analysis using two-way repeated measures analysis of variance (ANOVA), which estimates the differential change in the two treatments on the outcome measures. We also specified an error term to account for individual variation. Subsequently, data derived from the first IGF-1 study (n = 9) [9], which was also a randomized crossover design, were assembled with the current study (n = 10) to generate a larger pooled data set (N = 19). Using this pooled data set, we applied the same treatment × time interaction analysis while controlling for baseline measurements and data set effects as covariates. Finally, because of the small sample size and to cast a wide net of informative treatment effects at threshold significance level, we applied an exploratory approach implementing a Mann–Whitney U-test on fold-changes observed at week 12 for each treatment group using the combined sample (N = 19).

## Results

**Fig. 1:**
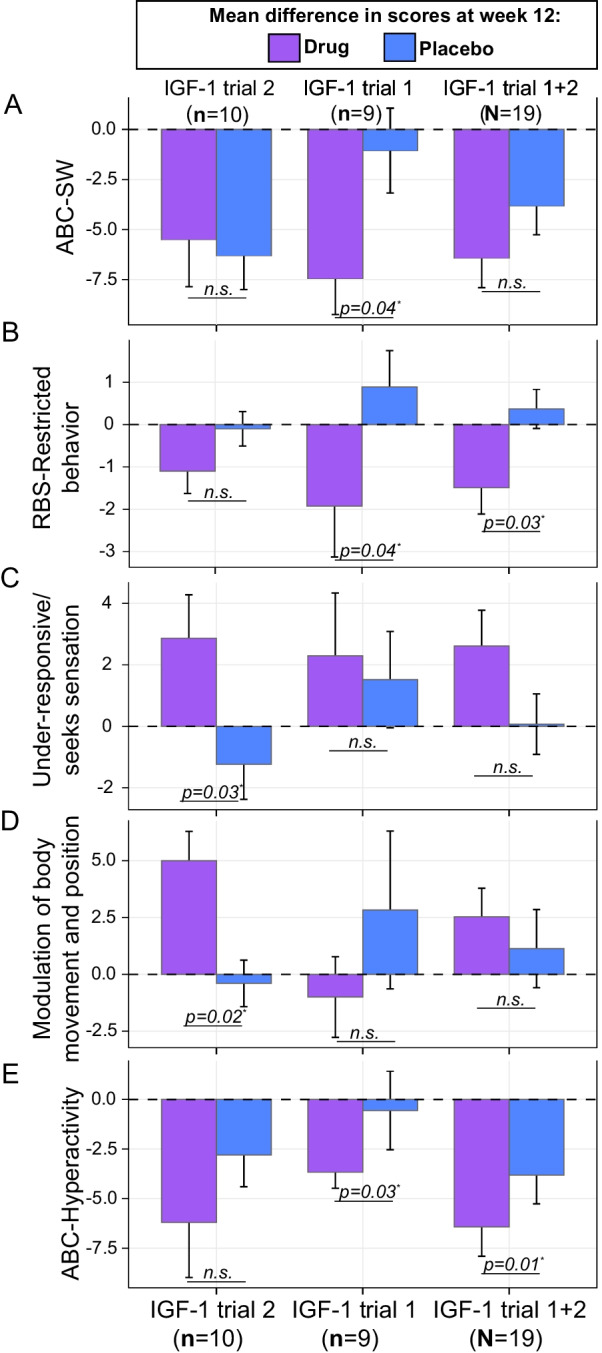
Significant outcome measures between baseline and week 12 of drug (IGF-1) or placebo. Mean changes are shown across the second study (left), first study [9] (center) and the combined effect (right) for the following significant outcome scores testing for time × treatment interactions: **A** ABC-SW, significant effect in the first study only (*p* = 0.04); **B** RBS-R Restricted Behavior, significant effect in the first study (*p* = 0.04) and combined study effect (*p* = 0.03); **C** SP Under-responsive/seeks sensation, significant effect in the second study only (*p* = 0.03); and **D** SP Modulation of body movement and position, significant effect in the second study only (*p* = 0.02). **E** Exploratory analysis applying a Mann Whitney-U test on difference in scores at 12 weeks identified significant reduction in ABC-hyperactivity scores in the first (*p* = 0.03) and combined study effect (*p* = 0.01). Mean differences and standard errors are shown for all outcome measures. Abbreviations: ABC-SW = Aberrant Behavior Checklist—Social Withdrawal subscale; RBS = Repetitive Behavior Scale; SP = Sensory Profile; ABC-hyperactivity = Aberrant Behavior Checklist-hyperactivity

There were no serious adverse events. Height, weight, neurologic, cardiac, bone age, and laboratory monitoring did not show any evidence of clinically significant changes. In this second study, hypoglycemia occurred in 3/10 patients for a total of 4 times while on IGF-1 and in 3/10 patients for a total of 9 times while on placebo. With the exception of one occurrence in one participant while on IGF-1, there were no clinical symptoms of hypoglycemia. The most common adverse events (AEs) during IGF-1 treatment were runny nose/congestion (n = 5), increased appetite (n = 5), lethargy/decreased energy (n = 5), and mood changes/irritability (n = 5). The number of adverse events reported was not significantly different between IGF-1 and placebo treatment arms (*p* = 0.635, Cohen’s d = 0.03) (Additional file 1: Table S1). In analysis combining results across both studies, there was a higher incidence of adverse effects in the IGF-1 treatment arm as compared to the placebo arm (*p* = 0.017, Cohen’s d = 0.23). Results on the primary outcome measure, the ABC-SW, did not reach statistical significance in the second study (*p* = 0.81) or combined dataset (*p* = 0.28) despite positive results from the first trial (*p* = 0.04) (Fig. 1A). The placebo response was higher in this second study (difference at 12 weeks: -6.3 ± 5.38) compared to the first study (difference at 12 weeks: − 1.5 ± 4.12), thereby eliminating the statistical separation between groups.

Improvement in repetitive behaviors with IGF-1 was not statistically significant in the second study (*p* = 0.08), however, results were consistently in the direction of improvement. Further, when we combined data from both studies, significant reductions on the Restricted Behavior subscale of the RBS-R were observed (*p* = 0.008) and aligned with results from the first trial (*p* = 0.04) (Fig. 1B).

In addition, we found improvement on two domains of the SP, a validated caregiver questionnaire measuring sensory reactivity where higher scores reflect more typical responses. Profound sensory under-responsiveness has been previously documented in PMS [21, 22]. In this second study, we found that the “sensory under-responsiveness/seeks sensation” domain derived from the Short Sensory Profile (SSP), as well as the related “modulation related to body movement and position” domain on the SP, both improved significantly with IGF-1 as compared to placebo (*p* = 0.03, *p* = 0.02, respectively) (Fig. 1C, D). Though the direction of effects was similar in both sets of combined study analyses, results only reached statistical significance in the new data set. There were no other statistically significant effects seen across other clinical outcome variables in the second study (Additional file 2: Table S2) or the combined effect across both studies.

Finally, we relaxed our statistical thresholds and applied an exploratory approach testing the mean changes observed at week 12 for each treatment group using the combined sample (*N* = 19). This approach revealed a new finding with respect to hyperactivity as measured by the ABC Hyperactivity subscale. Improvement in hyperactivity was sufficient to reach statistical significance when analyzing both studies jointly (*p* = 0.01) (Fig. 1E).

## Limitations

Results must be interpreted with caution given the small sample sizes of both studies and challenges inherent in combining datasets. In addition, relying on parent reported outcome measures may introduce expectancy bias that warrants caution in interpreting results.

## Conclusions

IGF-1 is safe over the course of 12 weeks of treatment and holds promise for treating symptoms of PMS, including social withdrawal, repetitive behaviors, hyperactivity, and sensory reactivity. The limited replication between this study and the previous with regard to social withdrawal symptoms may have occurred in part because the first study results were published before completing the second study, possibly biasing participants on this caregiver report measure. This study also presents new findings highlighting the potential impact of IGF-1 on sensory processing abnormalities, including sensory hyporeactivity, which is a common feature of PMS. Future efforts should employ larger sample sizes and conduct trials across multiple centers to ensure rigor. Further, other compounds that increase endogenous levels of IGF-1 should be considered and at least two small studies with growth hormone show promise [23, 24].

## Supplementary Information

**Additional file 1: Table S1.** Adverse events associated with IGF-1.

**Additional file 2: Table S2.** Clinical outcomes of second IGF-1 study (n = 10).

## Data Availability

The datasets used and/or analyzed during the current study are available from the corresponding author on reasonable request.

## Abbreviations

ABC: Aberrant Behavior Checklist
ASD: Autism spectrum disorder
CGI: Clinical Global Impression Scale
FDA: Food and Drug Administration
PMS: Phelan-McDermid syndrome
RBS-R: Repetitive Behavior Scale-Revised
SP: Sensory Profile
SSP: Short Sensory Profile
