# Supplementary material for: Clinical trial of insulin-like growth factor-1 in Phelan-McDermid syndrome
Source: Mol Autism. 2022 Apr 8;13:17. doi: 10.1186/s13229-022-00493-7 (PMC8994375; doi:10.1186/s13229-022-00493-7)
Supplement: Supplementary file 1 — Additional file 1: Table S1. Adverse events associated with IGF-1. [file 13229_2022_493_MOESM1_ESM.docx]

**Supplemental Table 1**. Adverse events associated with IGF-1

| **Adverse Event** | **IGF-1** | **Placebo** |  |
| --- | --- | --- | --- |
| Runny nose/congestion | 6 | 7 |  |
| Sleep disturbance | 4 | 4 |  |
| Mood changes/irritability | 5 | 6 |  |
| Broken bone | 0 | 1 |  |
| Upper respiratory tract infection | 4 | 5 |  |
| Lethargy/decreased energy | 5 | 3 |  |
| Cough | 2 | 2 |  |
| Redness around perineum | 1 | 0 |  |
| Increased appetite | 5 | 1 |  |
| Facial swelling | 1 | 0 |  |
| Diarrhea | 1 | 3 |  |
| Increased ear wax | 0 | 1 |  |
| Decreased appetite | 2 | 2 |  |
| Increased energy | 1 | 1 |  |
| Constipation | 3 | 1 |  |
| Gait changes/fall | 2 | 3 |  |
| Rash | 0 | 1 |  |
| Vomiting | 3 | 2 |  |
| Bloody nose | 1 | 2 |  |
| Increased reflux | 1 | 1 |  |
| Increased chewing/biting | 1 | 0 |  |
| Insomnia | 0 | 1 |  |
| Elevated heart rate | 0 | 1 |  |
| Fever | 4 | 2 |  |
| Choking on food | 1 | 0 |  |
| Ear infection | 1 | 1 |  |
| Seizure | 1 | 0 |  |
| Urinary tract infection | 0 | 1 |  |
| Bruising at injection site | 1 | 0 |  |
| Breast buds developed | 1 | 0 |  |
| Increased drooling | 1 | 0 |  |
| Increased aggression | 0 | 1 |  |
| Warmer body temperature | 1 | 1 |  |
| Stomach virus | 2 | 0 |  |
| Increased eye rolling | 1 | 0 |  |
| Stomach aches | 0 | 1 |  |
| Hypoglycemia* | 4 | 9 |  |
|  |  |  |  |
|  |  |  |  |

*Hypoglycemia occurred in 3/10 patients while on IGF-1 and in 3/10 patients while on placebo
