# Supplementary material for: Clinical trial of insulin-like growth factor-1 in Phelan-McDermid syndrome
Source: Mol Autism. 2022 Apr 8;13:17. doi: 10.1186/s13229-022-00493-7 (PMC8994375; doi:10.1186/s13229-022-00493-7)
Supplement: Supplementary file 2 — Additional file 2: Table S2. Clinical outcomes of second IGF-1 study (n = 10). [file 13229_2022_493_MOESM2_ESM.docx]

**Supplementary Table 2.** Clinical outcomes of second IGF-1 study (n=10)

|  | **Differences at 12 weeks (mean ± s.d.)** | | ***P*-value significance** | |
| --- | --- | --- | --- | --- |
| **Variable** | **WK12 Δ (Drug)** | **WK12 Δ  Placebo** | **Time ×** **treatment** | **Mann Whitney-U** |
| ABC-Irritability | -2.4 ± 4.6 | -3.8 ± 5.29 | 0.60 | 1.0 |
| ABC-Social Withdrawal | -5.5 ± 7.46 | -6.3 ± 5.38 | 0.81 | 0.65 |
| ABC-Sterotypy | -0.8 ± 2.49 | -2.6 ± 2.76 | 0.25 | 0.37 |
| ABC-Hyperactivity | -6.2 ± 8.78 | -2.8 ± 5.05 | 0.37 | 0.24 |
| ABC-Inappropriate Speech | -0.8 ± 1.55 | -1.9 ± 4.38 | 0.39 | 0.45 |
| CGI-Severity | -0.2 ± 0.42 | -0.1 ± 0.32 | 0.50 | 0.63 |
| CGI-Improvement | 2.6 ± 0.84 | 3.1 ± 1.28 | NA | 0.31 |
| RBS-Stereotyped Behavior | -0.3 ± 2.63 | -0.8 ± 2.19 | 0.69 | 0.76 |
| RBS-Self-Injurious Behavior | -0.3 ± 1.64 | 0.2 ± 1.14 | 0.54 | 0.41 |
| RBS-Compulsive Behavior | -0.8 ± 1.4 | 0.1 ± 0.88 | 0.13 | 0.18 |
| RBS-Sameness Behavior | -1.4 ± 2.27 | -0.37 ± 1.74 | 0.44 | 0.19 |
| RBS-Restricted Behavior | -1.1 ± 1.66 | -0.1 ± 1.29 | 0.08 | 0.29 |
| RBS-Total | -3.5 ± 6.2 | -0.77 ± 5.73 | 0.46 | 0.21 |
| SP-Auditory Processing | 1.6 ± 2.46 | 0.2 ± 2.39 | 0.25 | 0.31 |
| SP-Visual Processing | 1.3 ± 4.92 | 1.5 ± 3.69 | 0.92 | 0.97 |
| SP-Vestibular Processing | 0.9 ± 5.2 | -0.53 ± 4.06 | 0.49 | 0.79 |
| SP-Touch Processing | 3.9 ± 4.89 | 0.3 ± 5.36 | 0.23 | 0.17 |
| SP-Multisensory Processing | 0.9 ± 4.23 | -0.7 ± 3.68 | 0.38 | 0.73 |
| SP-Oral Sensory Processing | 1 ± 5.08 | 0.5 ± 5.32 | 0.83 | 0.79 |
| SP-Sensory Processing Related to Endurance/Tone | 0.5 ± 5.93 | 2 ± 3.33 | 0.57 | 0.71 |
| SP-Modulation Related to Body Position and Movement | 5 ± 4.08 | -0.4 ± 3.24 | 0.02* | 0.01* |
| SP-Modulation of Movement Affecting Activity Level | 0.2 ± 3.16 | 1.7 ± 5.58 | 0.50 | 0.57 |
| SP-Modulation of Sensory Input  Affecting Emotional Responses | -0.4 ± 1.17 | 0.6 ± 1.84 | 0.29 | 0.21 |
| SP-Modulation of Visual Input Affecting Emotional Responses  and Activity Level | 0.4 ± 1.65 | 0.9 ± 1.52 | 0.62 | 0.57 |
| SP-Emotional/Social Responses | 3 ± 7.85 | 0.1 ± 5.3 | 0.41 | 0.48 |
| SP-Behavioral Outcomes of  Sensory Processing | 2.83 ± 6.59 | 0.11 ± 3.55 | 0.23 | 0.55 |
| SP-Items Indicating Thresholds  for Response | 1 ± 1.55 | 0 ± 2 | 0.16 | 0.17 |
| SSP-Tactile Sensitivity | 0.9 ± 2.73 | 0.7 ± 2.45 | 0.88 | 0.85 |
| SSP-Taste/Smell Sensitivity | -0.2 ± 2.57 | -0.5 ± 1.08 | 0.76 | 0.17 |
| SSP-Movement Sensitivity | 0.4 ± 1.26 | -0.3 ± 1.57 | 0.39 | 0.32 |
| SSP-Underresponsive/Seeks Sensation | 2.87 ± 4.47 | -1.23 ± 3.61 | 0.03* | 0.04* |
| SSP-Auditory Filtering | 0.9 ± 2.23 | 0.3 ± 2.67 | 0.59 | 0.54 |
| SSP-Low Energy/Weak | -0.6 ± 3.89 | 1.36 ± 1.99 | 0.24 | 0.14 |
| SSP-Visual/Auditory Sensitivity | 0.8 ± 1.23 | 0.8 ± 1.55 | 1.0 | 0.88 |
| SSP-Total | 5.07 ± 11.43 | 1.13 ± 10.32 | 0.50 | 0.36 |
| A treatment × time interaction analysis using two-way repeated measures analysis of variance estimated the differential change in the two treatments on each variable. An exploratory analysis using a Mann Whitney-U test was applied to mean differences at 12 weeks between drug and placebo. | | | | |
